# Understanding the pay equity from the idea of universal equality in traditional Chinese philosophy

**DOI:** 10.3389/fpsyg.2022.998553

**Published:** 2022-10-20

**Authors:** Yuanjun Cui

**Affiliations:** Department of Philosophy, College of Political Science and Law, Capital Normal University, Beijing, China

**Keywords:** pay equity, social psychology, attitude, behavior, Chinese philosophy, universal equality

## Abstract

Pay equity is not only a manifestation of social behavior but also a reflection of gender attitude. In view of the close interactions between pay equity, gender attitude and cultural value, it is necessary to examine the social-psychological connotations behind the pay equity concept and to seek its theoretical basis and support at the philosophical level. By examining the main ideas in Chinese philosophy, this paper claims that traditional Chinese culture contains rich connotations related to the pay equity concept and the pursuit of gender equality. These ideas can not only shape implicit nondiscriminatory gender attitudes but also inhibit the male superiority and intergroup prejudice through the self-regulation mechanism. The collision between traditional ideological insights and the modern pluralistic society will certainly promote the renewal of gender equity and concomitant changes in social attitude and employment behavior.

## Introduction

Pay equity (also known as “pay equity” or “comparable value for work”) is generally deemed one of the earliest international labor standards established across the world. This principle was recognized in the founding statutes of the International Labor Organization (ILO) in 1919. The basic premise of pay equity emphasizes that different social groups should receive equal remuneration or the same level of remuneration when they engage in the same work or make equal contributions (Fawcett, 1918). The pay equity concept is mainly concentrated on female workers and labor dispatch workers. The reason why the status of this concept can be so widely accepted is that pay equity embodies the value implication and pursuit for equal employment opportunities and antidiscrimination among all workers. It calls for not only respect for the values of workers but also the maintenance of their rights. For a long time, extensive research has been carried out on the pay equity concept from legal, economic, and political science perspectives (e.g., Örtenblad, 2021), and explanations for the gender pay gap have come from a number of different disciplinary fields. For example, Rubery and Grimshaw distinguished among four distinct analytical approaches: economic, sociological, organizational and institutional (Rubery and Grimshaw, 2015). They argued that each approach focused on different factors as to the causes of inequality in men’s and women’s pay. These different causes implied different solutions as to how to reduce this gap. Mary Cornish, after examining legal developments and their impacts, suggested that pay equality strategies that only focused on the formal employment sectors should be restructured to establish workplace governance models by combining mandatory laws with existing norms, to design gender-sensitive international, regional, national and corporate-level pay equity programs and to develop multilayered pay strategies to incorporate the recognition of female workers into the mainstream evaluation system (Cornish, 2007). From the perspectives of value, system and event, Song examined how “labor” and “gender,” as two organizational methods, help female workers form their subjectivity in the labor process and analyzed the liberation and restriction of pay equity by considering female body–mind tension (Song, 2020).

However, the core of pay equity comprises the cognitive concepts of opposing prejudice and inequality and pursuing a reasonable and fair evaluation system. These values are deeply associated with social cognitive elements such as intergroup attitudes, biases, and stereotypes, but these related topics have rarely been discussed. This paper mainly focuses on the social-psychological connotations of the pay equity concept, aiming to seek its theoretical support from the perspectives of philosophy and cultural influence.

## Gender attitudes and culture

From the perspective of social psychology, the phenomenon of unequal pay for equal work is essentially an inappropriate evaluation of the capabilities of different workers caused by negative attitudes such as bias and discrimination based on gender and the resulting inequality in employment opportunities. A typical manifestation is that female workers who are engaged in the same labor content are often paid with lower labor remuneration rates or classified according to different remuneration standards. Attitude and behavior are central research topics in social psychology. Initially, most researchers focused on exploring the relationship between attitude and behavior to reveal the predictive power of attitude on behavior. In the past three decades, the research focus in the attitude–behavior field has gradually shifted to the moderating variables of the specific relationship between attitude and behavior. Accordingly, two independent research directions are formed. The first direction concerns the influence of third-party variables (such as subjective norms and perceived behavioral control) on the relationship between attitude and behavior (Leone et al., 1999), represented by the research on the theory of planned behavior (Ajzen, 1991). The second research direction concerns the influence of the characteristics of attitude on attitude and behavior (e.g., Crano, 1997). The proposal of the pay equity concept aims to correct the biased social phenomenon of unequal pay for equal work by formulating institutionalized arrangements and regulations (i.e., third-party variables above), but it lacks attention to the impact of the characteristics of attitude itself on social behavior.

With the emergence of fruitful research outcomes on attitude and the establishment of corresponding theories, most researchers agree that evaluation is the main element of attitude response, and once an attitude is formed, it can be expressed in two forms, i.e., explicit attitude and implicit attitude (the dual attitude model; Wilson et al., 2000). Explicit attitude refers to a kind of evaluation formed after thorough consideration and self-regulation, which can be easily reported. The measurement of explicit attitude is generally achieved by directly requesting individuals to report their evaluation of an object, which may be influenced by the deliberate response strategy under social expectations or self-expression. Explicit attitude is unable to capture the psychological content that fails to be obtained through introspection (e.g., Gawronski, 2009). In contrast, implicit attitude is often deemed to be created by automatic, uncontrolled associative processing. The measurement of implicit attitude does not require respondents to directly report their thoughts and beliefs so that problems such as “unwillingness and inability” can be minimized to the greatest extent. Therefore, the implicit attitude sometimes runs counter to an individual’s intentional, thoughtful, and socially approved explicit attitude. For example, a society may hold negative implicit attitudes toward women, but its explicit attitudes toward women can be positive.

One point of view argues that implicit attitudes can be influenced by sociocultural knowledge, and such knowledge is different from attitude (Karpinski and Hilton, 2001). However, another point of view stresses that implicit attitudes reflect the accumulation of experiences. These experiences may not be used for introspection and may not be needed or socially recognized but are still attitudinal, as they can influence an individual’s perception, judgment or action (Banaji et al., 2004). Although the above two points of view seem to be contradictory based on the composition of attitude, neither of them denies the common influence of cultural variables on dual attitudes.

## Chinese cultural resources for the idea of equality

Relying solely on institutional constraints and restrictions to improve cognitive concepts may not be sufficient to reach an ideal state. Therefore, it appears to be necessary to explore the attitudinal components of pay equity and to seek corresponding theoretical support at the cultural level. Ancient Chinese philosophy contains rich connotations on the idea of equality and respects all things equally, including human beings. Examining the idea of equality in traditional Chinese philosophy may play a guiding role in interpreting the pay equity concept to promote the widespread recognition of this concept in modern society.

It is generally understood that Chinese culture attaches great importance to the hierarchical social structure and emphasizes the difference between the superior and the inferior. In fact, ancient Chinese philosophy, represented by the three schools of Confucianism, Mohism and Taoism, contains the idea of equality to varying degrees. The Confucian concept of equality can be traced back to *The I Ching* (James, 1963). The Six Classics, i.e., *Book of Poetry, Book of History, The Etiquette, The I Ching, Spring and Autumn*, and *Book of Music*, are generally thought to have been edited by Confucius, while the *The I Ching · The Great Appendix* was written by Confucius’s students. Altogether, these classic books constitute the primary historical literature of Confucian thought. A most prominent characteristic of *The I Ching* is that it classifies the various complex natural and social phenomena in the universe into two abstract categories—“Yin” and “Yang”—which are used to summarize and reveal the most basic laws of all things and the internal reasons for the development of things. In *The I Ching*, Yin and Yang are considered two equal categories that are complementary to each other, representing different characteristics in the universe. The sympathetic interaction between Yin and Yang is the inner driving force and the fundamental reason for the development of things. *The I Ching · The Great Appendix* emphasizes that Yin and Yang together form “Tao” (In Chinese: 一阴一阳之谓道), which means that the highest noumenon of Tao is composed of both Yin and Yang – both are indispensable. In Confucian philosophy, Yin and Yang are two equally important elements with equal status. In real-world society, Yin and Yang often correspond to the relationship between men and women. In fact, in the opinion of some Confucian philosophers, the status of women is even slightly higher than that of men. For example, with respect to the “Xian” hexagram of the *The I Ching*, a famous Confucian figure, Hsun Tzu, commented that the improvement of women’s status was a symbol of auspiciousness (In Chinese: 以高下下，以男下女，柔上而刚下; John, 1988).

From this point of view, the idea of equality in Confucianism has a clear origin, and early Confucian representatives generally attached great importance to the status of women. Their focus of thinking was mainly rooted in family ethics, but this does not necessarily mean that the founders of Confucianism intended to confine women to family matters only. A possible explanation is that Confucianism regards family as the foundation of the world and argues that the governance of a country must start from the governance of a family. Unfortunately, after Dong Zhongshu of the Han Dynasty “exclusively” recognized Confucianism, some Confucian ideas were politically exploited and misinterpreted – one such misinterpretation is that women should be imprisoned under the authority of the husband and become the vassal of men (such as the well-known “Three Principles and Five Virtues,”in Chinese: 三纲五常). Nevertheless, the original meaning of Confucian thought should not be denied because it was misinterpreted and politically exploited. Since early Confucianism neither deliberately lowered the status of women nor held any prejudice against women, women were an important labor force during the Warring States Period and had equal status to that of male laborers. In the classic book *Kaogongji*, which records the methods used by handicraft workers in making utensils and tools in the Warring States Period, six major occupations covering all social groups in ancient Chinese society (In Chinese: 国有六职; see Feng, 1991), from the senior ruling class down to the underclass of peasants, were defined, including “handicraft workers,” “merchants,” “peasants,” “female workers” and “aristocrats.” It is worth noting that “female workers,” which refer to women who were engaged in knitting production, were regarded as one of the six parallel occupational groups. Similar to the governors of the country, all six occupational groups were considered important components of the national economy. This kind of occupational division system affirmed the labor rights and the rights to receive labor remuneration for different labor groups, including women. The above content in *Kaogongji* shows that during the Spring and Autumn Period and the Warring States Period of ancient China, women were equal to men in social status and in labor remuneration. In another classic book during the Warring States Period, *Mo Zi*, it was proposed to determine the labor remuneration rate based on labor conditions and amount of contributions (In Chinese: 以劳殿赏，量功而分禄; Ian, 2010). In addition, other statements in *Mo Zi* express objections against treating different social groups or classes unequally, such as “promoting all capable people even though they are peasants or handcraft workers” (In Chinese: 虽在农与工肆之人，有能则举之; Ian, 2010) and “not to treat people differently due to differences in wealth or social status”(In Chinese: 虽天亦不辩贫富、贵贱、远近、亲疏) (Ian, 2010). Although the historical literature handed down by the Mohists is limited, we can still trace a strong and clear ideology of pursuing equality in Mohist thought.

Compared with the seemingly latent ideas of equality in Confucianism and Mohism, the Taoist idea of equality is much richer and more philosophical. Taoist thought looks at group differences from the levels of cosmology and world view and proposes to treat all kinds of social groups and even all things equally according to the concept of “transcendence.” Taoist philosophy takes Tao as the highest noumenon, believing that the effect of Tao on all things in the universe occurs naturally. The relationship between Tao and all things is “to create” and “to be created.” However, although Tao has such an extraordinary meaning and effect on all things, it does not override anything. In contrast, Tao holds an equal attitude toward all things, allowing them to self-cultivate and self-complete under natural conditions. *Lao Tzu* argued that Tao did not have any partiality or preference for all things in the universe (In Chinese: 天地不仁，以万物为刍狗; Arthur, 1934), which means that, in front of Tao, all things are treated equally. It is worth noting that in the thought conveyed by *Lao Tzu*, human beings are regarded as part of all things, so Tao treats human beings with no partiality and no preference compared to any other creatures and objects. When this idea is applied to the relationships between different groups in real-world society, it means that all individuals and all social groups should be treated equally in front of Tao, with no difference or discrimination. The relationships between different individuals and between different groups would therefore transcend the actual variations in reality and result in the fairest treatment.

All three main theories of Chinese philosophy, i.e., Confucianism, Mohism, and Taoism, have discussed the topic of equality among individuals. They commonly agree that factors like gender, wealth and social status should not affect the equal relationship among different people. However, confucianism, starting from the internal causes of the development of things, advocates treating two objects that are opposite to each other equally, and believes that men and women form an equal relationship similar to the complementation between Yin and Yang. Mohism claims that workers have equal status from the perspective of social reality. Taoism, from the perspective of cosmology, argues that all things in the universe are equal. Perhaps it is because of these ideas, deeply rooted in Chinese philosophy, that in the later Ming and Qing Dynasties, it can still be seen from the literature records that women did not have a significantly lower status than men in terms of labor conditions and labor remuneration, despite the fact that women were seriously imprisoned and restricted by the feudal ideology. Fan Lian’s *Yunjian Jumiao* recorded that all men and women in Songjiang County made socks for a living, and their labor remuneration was paid based on the actual value (In Chinese: 从店中给酬取值; see Tong, 1981). In *Zhengyang (Henan) County Chronicle · Production Chronicle* during the Jiaqing period of the Qing Dynasty, it was recorded that all families in Zhengyang County had machines at home, and both men and women were involved in production (In Chinese: 家家设机，男女操作; see Liu and Hou, 2005). This implied that women and men played equal occupational roles at that time. Moreover, in the Ming and Qing Dynasties, the breadth of women’s employment was also surprisingly wide. According to historical literature, a woman in Huzhou was engaged in the profession of law and was said to “make huge profit due to her outstanding professional abilities” (In Chinese: 刀笔为讼师…凭其一字数笔…因之射利; see Zeng, 2004). This record may well demonstrate that female practitioners were not inferior to men in terms of labor conditions and remuneration rate at that time. There are many other similar records and evidence in the literature, but due to space limitations, only a few examples are listed in this paper.

## Pay equity, gender attitude, and universal equality

Even Taoism, which attaches great importance to universal equality, admits that differences in human society exist objectively. Gender, blood relationship, and age can all be regarded as criteria for group division and can be seen as differences between individuals. However, the existence of differences does not necessarily lead to prejudice or bias against any particular group. The root cause of prejudice or bias lies in the cognitive concept based on which differences are treated. It can be inferred from many previous research that the universal equality idea in Chinese philosophy can not only shape implicit nondiscriminatory gender attitudes but also inhibit the intragroup superiority and intergroup prejudice through the self-regulation mechanism of attitudes (Bagozzi et al., 2003; Matsumoto et al., 2008). For example, Greenwald et al. (1998: experiment) found that Korean and Japanese American students showed greater automatic in-group bias to the extent they were immersed in their ancestors’ cultural knowledge. Further analysis based on multinomial modeling suggested that these effects may be related to inhibition ability of biased associations inherited from cultural values, but not to changing the associations that were activated (Allen et al., 2010).

It seems plausible that culture is a critical variable in the process of shaping pay equity with gender attitude. In a nutshell, regardless of the mechanism through which gender attitude is expressed, it is influenced by the culture.

In our general understanding, men are superior to women in traditional Chinese culture, and husbands are even regarded as the guide for wives. As women are often deemed representatives of inferiority, it may not be easy to imagine that there are distinct concepts supporting gender equality and pay equity in the traditional Chinese philosophical system and cultural value. A comprehensive evaluation of the main ideas in historical evidence and literature revealed that Chinese culture actually contained rich connotations related to the pay equity concept and the pursuit of gender equality. The equality idea in traditional Chinese philosophy provides human beings with valuable ideological resources. Such a strong sense of equality has a high degree of relevance to the cognitive awareness pursued by the pay equity concept. At the same time, because of its relatively sound and sophisticated logical reasoning, the equality idea in Chinese philosophy can also provide philosophical support for the highly realistic concept of pay equity to open up a logical path for the further development of this concept from a philosophical perspective. The collision between the traditional ideological insights and the modern pluralistic society will certainly promote the renewal of gender equity and concomitant employment behaviors and social attitudes and facilitate the realization of pay equity between men and women in China and even across Asia.

## Data availability statement

The original contributions presented in the study are included in the article/supplementary material, further inquiries can be directed to the corresponding author.

## Author contributions

The author confirms being the sole contributor of this work and has approved it for publication.

## Conflict of interest

The author declares that the research was conducted in the absence of any commercial or financial relationships that could be construed as a potential conflict of interest.

## Publisher’s note

All claims expressed in this article are solely those of the authors and do not necessarily represent those of their affiliated organizations, or those of the publisher, the editors and the reviewers. Any product that may be evaluated in this article, or claim that may be made by its manufacturer, is not guaranteed or endorsed by the publisher.
